# A Case of Carcinoma Ventriculi

**Published:** 1879-11

**Authors:** E. G. Zinke

**Affiliations:** Assistant to the Chair of Gynæcology, Medical College of Ohio


### A CASE OF CARCINOMA VENTRICULI.

By E. G. ZINKE, M.D.,

Assistant to the Chair of Gynæcology, Medical College of Ohio.

The subject of my remarks for this evening is a case which came under my observation about February 1st, 1879. The patient, Mr. S—, æt. 49, a tinner by trade, was a tall and well developed individual; he was also a man respected in the community for his steadiness of character and regular habits, not dissipated or addicted to the use of drink at any time during his life. When he presented himself to me, although still well nourished, he bore the countenance of internal suffering; he was pale, the alæ of the nose and naso-labial folds were deep and sharply defined, and the rest of his features completed the index of a lingering affection. On inquiry he stated that for the last year and more he had suffered with dyspepsia, gradual loss of flesh and vigor, but not enough to disable him from work. Physical examination developed nothing except a slight cardiac bruit, which I attributed to anæmia so evident in his outward appearance. He had no pain, no fever, and his family history suggested nothing of importance.

*Diagnosis:* Chronic catarrh of the stomach. He was directed to live principally upon milk, (Koumiss to be used quite freely,) meat-broths, etc., and took tr. nucis vomicæ gtts. v. before, and liq. pot. ars, gtts. iv. after each meal. As his appetite improved he was allowed meats and such other articles as he might desire. Under this treatment he did well for about a month, when he had a relapse.

From that time on patient never recovered his appetite entirely, he felt better one day and worse the next, losing strength until about the end of March, a tumor was found in the abdominal cavity. The growth was hard and pulsating ; it was as large as a good-sized hen's egg, smooth and slightly modular, and located about one inch above the umbilicus and two inches to the left of the median line. It remained in position during the respiratory movements, but could be moved by the hand, as if attached by a pedicle to the spine ; pulsation ceased in knee-elbow position ; purcussion note dull, tympanitic. Repeated examinations showed that the tumor shifted about in the cavity ; one day it would be felt to the right and below the umbilicus ; the next day, perhaps, immediately above it ; then again below the same or in the region where first discovered ; towards the last of his life it remained fixed in the epigastrium, midway between the ensiform cartilage and umbilicus, commencing an inch to the right of the median line and extending to the junction of the epigastric with the left hypochondriac region. Soon after the appearance of the tumor, patient was confined to home and bed, he sank rapidly and suffered with increased pain in the region of the growth. He never vomited the medicine, and the food he took was apparently well borne until near his end it "laid heavily upon his stomach," using his own words, causing him some discomfort, but he never "threw it up." He died suddenly August 11th, during the act of vomiting and under the influence of morphine which had been administered in large doses to relieve him of his pain.

A post-mortem examination was made and cancer of the pylorus was found : The left lobe of the liver, pancreas and omentum showed extensive signs of secondary cancerous degeneration. Cancer of the mensentery was the diagnosis prior to patient's death. The post-mortem revelation was no surprise since it was deemed possible before, but was partially excluded on account of the absence of vomiting and the extreme mobility of the tumor within the abdominal cavity. It is needless to attempt to justify the error in diagnosis, because everyone knows how difficult it is to determine upon the true nature and location of an abdominal tumor in the male. This case simply furnishes additional proof of the already established fact that the absence of vomiting and the presence of a floating tumor in the abdomen do not preclude gastric cancer; the absence of the former may occur when the pyloric orifice has remained large enough to permit the passage of the ingesta (this specimen affords an illustration, its orifice is sufficiently ample to admit my index finger;) the presence of the latter is explained in that the tumor by reason of its weight drags the pylorus out of normal position and influenced by other circumstances may show itself in any of the abdominal regions. A case is reported where the tumor was found as low as the symphisis pubis.

Permit me to present to you the specimen removed from this patient. Its microscopic appearance is that of a large swelling around the pylorus extending nearly four inches along the lesser and greater curvature and anterior and posterior walls; it is almost one inch in thickness, hard to the touch and all the coats have been destroyed, excepting the serous which is also invaded as shown by the small heaps of morbid matter deposited within its substance. On the inner surface exuberent growths of soft, ragged and easily broken down masses can be seen. I have also here a microscopic section made throughout the whole thickness of the tumor, showing that not a trace of the coats proper is left, and that the connective tissue development is greatest toward the external, and the proliferation of cells greatest toward the internal surface Near the outer surface the characteristic alveoli, with the epithelial cells crowded into them, can be distinctly seen, while, the nearer we approach the inner surface only a mass of cells with but little connective tissue between them can be noticed.

Leub (Ziemmsen) and Niemeyer (Seitz) speak of three varieties of cancer of the stomach: 1st. carcinoma fibrosa or fibrous cancer (scirrhus). 2d. carcinoma medullare, or medullary cencer. 3d. carcinoma colloides, or alveola or gelatinoid cancer. The chief difference between the first and second is, that in the former the connective tissue element predominates over that of the cellular, in the latter the cellular element exists in greater abundance than the connective tissue. The third variety, colloid (gelatinous or alveola) has the same origin as the first two, but during its future development the accumulated cells within the alveoli undergo a gelatinous degeneration.

According to Waldeyer every cancer of the stomach springs from the epithelium lining the ducts of the mucous glands of this organ. The proliforation of these cells takes its way into the deeper structure of the stomach and thus the sub-mucous and muscular coats are first invaded and destroyed, while the mucous and serous layers give way to the disease process at a later day; the serous layer being the last attacked because of its lying furthest from the origin of the disease and its greater resistency to the same.

The fibrous and medullary cancers of the stomach are much more frequent than the colloid cancer. The pylorus is the part preferred by the malignant growth. According to Brinton, cancer of the pylorus occurs oftener than of the cardia, greater and lesser curvature, and anterior and posterior walls taken together.—*Cin. Lancet and Clinic.*

